# A Dual-Successive-Screen Model at Pollen/Stigma and Pollen Tube/Ovary Explaining Paradoxical Self-Incompatibility Diagnosis in the Olive Tree—An Interpretative Update of the Literature

**DOI:** 10.3390/plants10091938

**Published:** 2021-09-17

**Authors:** Catherine Marie Breton, Daniela Farinelli, Georgios Koubouris, Franco Famiani, Michel Raymond, André Bervillé

**Affiliations:** 1 Bioversity, Parc Scientifique Agropolis II, 1990 Boulevard de la Lironde, 34397 Montpellier, France; 2 Dipartimento di Scienze Agrarie, Alimentari e Ambientali (DSA3), Università degli Studi di Perugia, Via Borgo XX Giugno 74, 06121 Perugia, Italy; daniela.farinelli@unipg.it (D.F.); franco.famiani@unipg.it (F.F.); 3 ELGO-DIMITRA, Institute for Olive Tree, Subtropical Crops and Viticulture, Leoforos Karamanli 167, 73134 Chania, Greece; koubouris@elgo.iosv.gr; 4 Institut des Sciences de l’Evolution, CNRS, IRD, EPHE, Université de Montpellier, CEDEX 5, 34095 Montpellier, France; michel.raymond@umontpellier.fr; 5 INRA, UMR-DIAPC 1097, Supagro Bat 22, CEDEX 1, 34060 Montpellier, France; andre.jp.berville@orange.fr

**Keywords:** *Olea europaea* L., paleoploidy, sporophytic self-incompatibility, two S-loci

## Abstract

The ‘pollen test’ and ‘fruit set test’ following controlled crossing combinations of parents are the most commonly used methods for pollination incompatibility studies in *Olea europaea L*. Self-incompatibility (SI), with diagnoses based on the pollen test and pollen germination, indicating self-compatibility, is not always followed by fruit set in this species. To solve this dispute, we have reconciled all observations into a new model. Mismatches between field and laboratory data and between methods are resolved by the dual-successive-screen model (DSSM) supposing two different loci for the expression of the two SI mechanisms. Pollen/stigma is controlled by diallelic SI, or DSI, inferring two G1 and G2 compatibility/incompatibility (C/I) groups for varieties, whereas pollen tubes in ovaries are controlled by poly-allelic SI or PASI with twenty C/I groups. To explain the selfing of varieties, we have suggested that some determinants in the pollen tube and stigma are unstable and degrade (DS-D for degradation of S-determinant) after three to five days, enabling some pollen tubes to avoid being rejected, hence reaching ovules. DSI and PASI in the DSSM and DS-D mechanisms, plus the andromonoecy of the olive tree, complexify SI studies. Inferences from DSSM and DS-D mechanisms in olive orchard practice are detailed.

## 1. Introduction

Self-incompatibility (SI) is a general term including several mechanisms in angiosperms that prevent selfing and, thus, favor outcrossing and allogamy. Self-crossing is sometimes prevented physically due to the presence of distinct floral morphs, usually in the form of heterostyly, but SI genetic mechanisms may also be active when a pollen grain lands on a stigma of the same plant. The processes of pollen germination, pollen-tube growth, ovule fecundation, and embryo development are halted at one of its stages and, consequently, no fruit is produced [1]. SI is explained at a single S-locus, with different expression systems. The prevalent type of SI is gametophytic SI (GSI): the SI phenotype of the pollen is encoded in its own gametophytic haploid genome, whereas the two determinants are present in the stigma [1]. Due to expression after meiosis of the genes for each determinant in microspores, each individual produces two types of pollen grains. In sporophytic SI (SSI), the SI phenotype of the pollen is determined by the diploid genotype of the anther (the sporophyte). Determinants are expressed early in pollen mother cells, with eventual dominance relationships between the two S-alleles, and so each individual produces one type of pollen grain. The stigma usually harbors both determinants, but dominance relationships can also mitigate their expression [2]. In natural populations, a high number of alleles at the SI locus increases the probability that a nonself-pollen germinates, grows, and fertilizes an ovule, with the end result of producing a fruit with seeds [3]. Breeding practices have eliminated SI in most annual crops that are nowadays self-pollinated. For example, in domesticated Solanaceae, such as tomato, pepper, and eggplant, although their wild ancestors were self-incompatible, fruit production in modern varieties results from self-fertilization. In contrast, SI is still present in fruit crops [3]. In Rosaceae, for example, apple and pear [4], apricot [5], and almond [6] that share GSI, pairs of compatible varieties are sold in nurseries. In hazelnut (Betulaceae), SSI does not hamper fruit production, as the number of S-alleles is high with thirty-three S-alleles available in cultivated genotypes, favoring cross-pollination and sufficient fruit setting [7]. From an agronomical point of view, knowledge of the SI expression system in a given fruit crop, GSI or SSI, makes it possible to identify and predict compatibility or incompatibility in any pair of varieties.

Microscopic observations of pollen germination in vivo (herein referred to as ‘pollen test’) and the determination of fruit set (herein referred as ‘fruit set test’) following controlled crossing of the pairwise combinations of parents are the most common methods used for pollination incompatibility studies in *Olea europaea* L. Different methods have often led to contrasting conclusions about the diagnosis of compatibility/incompatibility (C/I) when pollen germination is observed on the stigma, but is not followed by fruit setting. Consequently, if self-incompatibility (SI) can be diagnosed by pollen testing and pollen germination, indicating self-compatibility, it is not always followed by fruit set in this species.

The SI mechanism of the olive tree was initially suggested to be gametophytic, until 2012, where it was shown that it is better explained as a sporophytic type [3,8,9]. This change was the result of di-allele crosses, showing that incompatible crosses do not always carry the same S-allele pair [9,10,11]. Thus, it is now generally agreed that an SSI mechanism operates in the olive tree [12], and that previous crossing results were inadequately interpreted, considering that partial results from SSI may match the GSI model [1]. A second puzzling feature of the olive reproductive system is that the number of S-alleles and the C/I groups are still unclear. For some authors, only two S-alleles are present, as shown by pollen test experiments, leading to two C/I classes, named G1 and G2 [12], although previous work, based on fruit setting experiments, has suggested higher numbers [9,10,11].

Based on pollen germination tests, another SSI mechanism was proposed as each variety clusters either in G1 (the S-allele pair is S1S2) or in G2 (the S-allele pair is S1S1). Pollen germination (compatibility) was observed in crosses of female G1 × male G2 and of female G2 × male G1. Then, if only two C/I groups exist, for example, the varieties (names in italics) *Picholine* and *Cayon* are incompatible when crossed in both directions, thus belonging to the same C/I group A. *Picholine* cannot pollinate *Tanche* and, thus, *Picholine* and *Tanche* belong to the same C/I group A. It is therefore expected that *Cayon* and *Tanche* are incompatible, because both are A; however, *Cayon* is an efficient pollinizer for *Tanche*; thus, *Tanche* and *Cayon* must belong to different C/I groups [9]. Thus, the use of only two C/I groups is insufficient to explain the data observed in the literature [8,9,11]. Crosses with some other varieties, such as *Aglandau*, *Frantoio*, *Grossane*, *Salonenque*, *Koroneiki*, and *Sevillano*, were recently analyzed with the result that the number of C/I groups increased [12]. Lastly, fruit setting occurring several days after pollination has been reported [12], suggesting that the pistil remains receptive, and that pollen tubes were blocked, but not killed, enabling late fertilization [13]. Thus, despite the fact that there is more than one century of data from crosses using a large number of varieties, and despite the consensus of the SI mechanism (SSI) since 2012, the number of SI alleles, and consequently the number of C/I groups, is still unsettled.

In the present review, in order to increase our knowledge in the controversial Olive SI, our approach is conceptual because two ways for analyzing SI in the olive lead to the proposal of two mechanisms. We gathered cross data from the literature to evaluate the occurrence of di-allelic self-incompatibility (DSI) and poly-allelic SI (PASI), and considered how to explain the presence of selfing in some varieties. As a result of this evaluation and comprehensive interpretation of data reported in the literature, we proposed a new hypothesis-model on the control of SI, which includes both mechanisms and is able to conciliate most of the various results available in the literature.

## 2. The Case of Olive Is Puzzling

### 2.1. Four Factors Might Explain the Difficulties

#### 2.1.1. Difficulties Due to Methods

**Figure 1:**
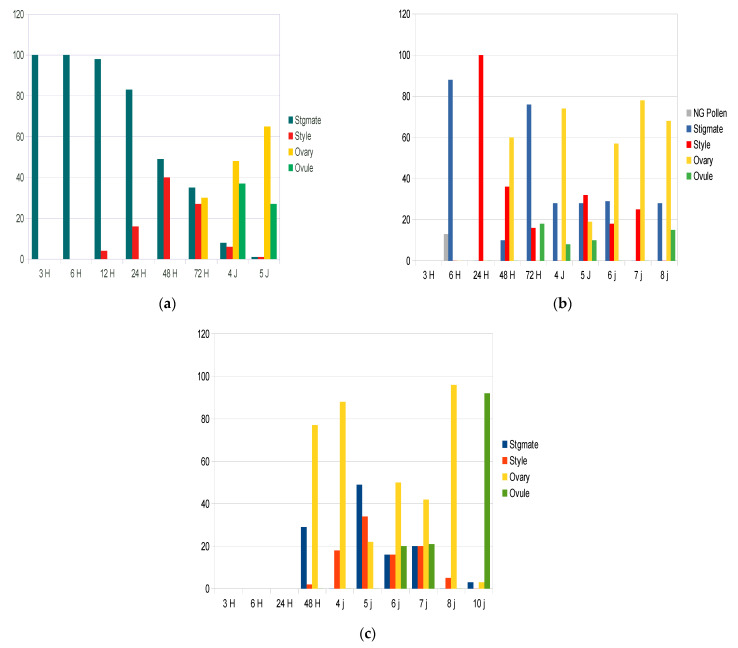
Histogram showing progression of pollen tubes over 10 days in (**a**) *Olivière* × *Arbequina*; (**b**) *Olivière* × *Chemlal*; (**c**) *Chemlal* × *Arbequina*. Redrawn from Ouksili [18].

**Figure 2:**
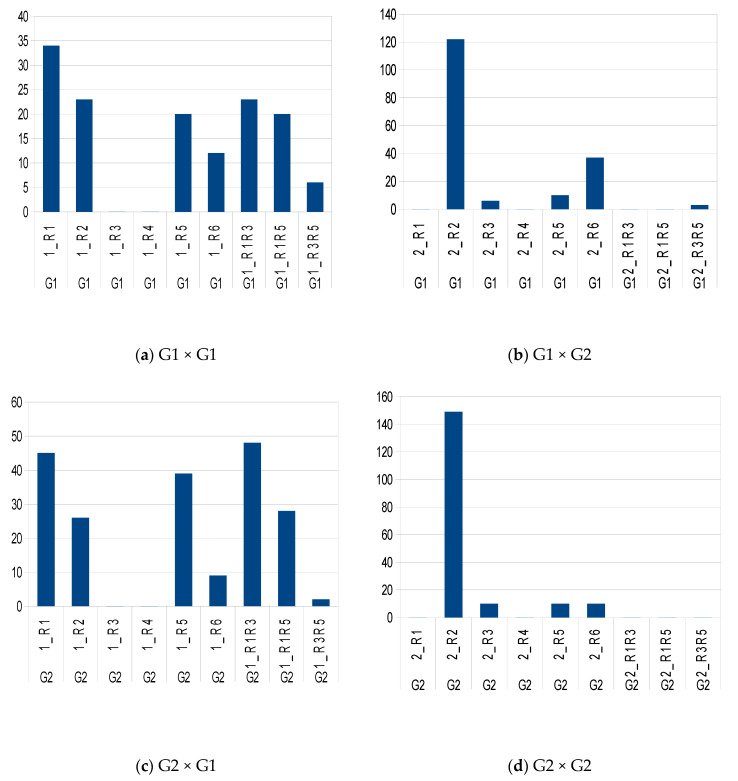
Number of crosses examined from the literature for pairwise combinations of varieties belonging to the frame of DSI to (**b**) G1 × G2 and (**c**) G2 × G1 expected with fruit in accordance with DSI and (**a**) G1 × G1 and (**d**) G2 × G2 expected without fruit, but that showed fruit, which sustained degradation of the S-determinants (DS-D).

The two methods used for assessing C/I, the in vivo pollen germination test and fruit set test, could provide different results. Pollen germination tests are widely used to experiment on SI in many species [1,12]: the growth of the pollen tubes close to the base of the stigma and entrance of the style is followed, usually until the pollen tube reaches the bottom of the style, but not further, because it is thought that nothing can prevent fertilization at this stage [2,3,12]. For a fruit test, foreign pollen is deposited onto the stigma of flowers protected from airborne pollen, at least 2 days before anthesis of its own pollen, and fruits are counted six weeks later. As a control, some inflorescences are protected from airborne pollen in order to estimate fruit set under selfing [14]. Presently, the model for SI in plants, in all studies and publications reported so far [2], asserts that the pollen grain that lands on the stigma immediately encounters the incompatibility barrier. The rapid response of the stigma to incompatible pollen has been widely documented in different species of Solanaceae, Rosaceae, and Papaveraceae for GSI, and in Brassicaceae, Betulaceae, and Asteraceae for SSI [7,15]. The self-incompatibility reaction is triggered in a few minutes or hours after self-pollen contacts the stigma [2,7,16], but can be as fast as one second in Papaveraceae [15]. However, exceptions such as late-acting self-incompatibility [1] have been encountered in different plant families. Some inconsistencies in the olive are reported in Table S1. However, pollen and fruit tests are equivalent if the SI control system operates only at the level of the stigma, which is considered as the norm for SI [1]. If the pollen germinates, thus indicating that it has a compatible sporophytic genotype, there are no further constraints to grow in the style and to reach an ovule for fertilization to occur [12]. As a consequence, the pollen germination test is predictive of the fruit set test, as is the case, for example, for hazelnut [7]. If this assumption is not correct, these two types of tests lead to inconsistent results. It has been documented that properties of pollen cell walls are modified not only along the pollen tube, but also in interaction to guidance cues inside the pistil [17]. For example, the C/I of pairwise combinations of olive varieties such as *Sevillano*, *Manzanillo*, and *Ascolano* cannot be deduced logically from pollen tests, because in some combinations of crosses, pollen tubes are arrested after reaching the base of the style, probably at the ovary level [8,13,16,18]. It has been shown that—the female is given first—in *Olivière* ♀ × *Arbequina* ♂ and in *Olivière* × *Chemlal* and *Olivière* × *Belgentier*, pollen tubes reach ovules from five to ten days after flower opening, respectively (Figure 1a–c) [18]. Thus, it is generally agreed that pollen germination tests are not to be trusted when diagnosing SI in comparison to fruit set, although some authors disagree [12]. Furthermore, Table 1 and Table 2 display crosses G1 × G1 and G2 × G2, respectively, reported to have produced fruits, although expected to provide no fruit, respectively [10,11,18,19,20,21,22,23,24,25,26,27,28,29]. However, 178 and 197 were of types G1 × G2 and G2 × G1 (thus expected to provide fruit) [8,12,30,31,32,33,34,35,36,37,38,39], respectively, and 138 and 179 of types G1 × G1 and G2 × G2 (thus expected to provide no fruit), respectively. It is shown that regardless of the PASI allele pair, for G1 × G1, all pollen classes have led to fruit on different hosts. The prevalent pollen class was R2 (92%) and a few for R3, R5 and R6 classes (Figure 2).

#### 2.1.2. Difficulties Due to Dominance Relationships between S-alleles

It has been shown that S-alleles display dominance relationships that are sex-specific [9]. Not considering these complex interactions may lead to incoherent results. Attribution of a pair of S-alleles to each variety involved in a di-allele cross was carried out [9], also considering a dominance relationship between the two S-alleles (Table 3), as was performed in Guayule [40]. Dominance relationships were separately established in the female part and in the male part due to the patterns of expression of the S-alleles [9,11]. In the pistil, all six S-alleles are codominant in pollen or pollen tubes: the transitive dominance relationships *R6* > *R2* > *R1* = *R3* = *R5* > *R4*, where dominance (>) and co-dominance (=) are found (Table 1). Such dominance/codominance relationships may change with additional crosses and possible new S-alleles may be described. For example, the variety *Picholine* has the genotype *R1R3* (and its pollen has the R1R3 codominant phenotype) as *Picholine* cannot fertilize *Cayon* (stigma phenotype [R1R4]) and *Tanche* (Stigma [R2R3]), whereas *Cayon* (pollen R1) cannot fertilize *Picholine*, because of its stigma [R1], but can fertilize *Tanche*, which does not carry the R1 allele. This explains that some pairs of varieties lead to symmetric crosses (crosses in the two directions provide similar results) for fruit setting due to codominant S-alleles, and some pairs of varieties with asymmetry (fruit in one direction and no fruit in the other direction) [6,8], due to the dominance of one S-allele over the other in the pair (Tables S1 and S2). A minimum of six S-alleles are required to explain cross-combinations from 102 varieties [8]. In any case, knowledge of the S-allele pair enables us to predict fruit set for crosses not yet performed between two varieties holding known pairs.

#### 2.1.3. Difficulties Due to the Number of Varieties Studied Is Insufficient

Third, the number of varieties used in published crosses is usually insufficient to identify the C/I groups. Most studies have dealt with two to four varieties except those of Moutier [22], 16 varieties, and Farinelli et al. [11], 26 varieties. Furthermore, most studies did not report pairs of varieties showing asymmetry, so it was not possible to identify C/I groups. The larger the number of varieties considered [10,11,41], the easier it is to identify C/I groups. In addition, the use of local varieties leads to C/I groups that are not readily comparable across studies from different countries. This is motivated by the economic importance of some varieties at a local level, but crosses using varieties planted on a large scale in different countries are more useful for collectively building a global list of C/I groups [14].

#### 2.1.4. Difficulties Due to Confusion between Selfing and Crosses

All SI models exclude the ability of selfing. It has been shown that this ability in olive varieties is common under bags [13,14,22,23,41], so a mechanism supposing that determinants may degrade with time along the transmitting tract tissue from stigma to ovary was proposed that did not impair the model for pollen tube advance [42,43]. Varieties used to obtain mapping progeny were crossed under bags, such as *Leccino* (1-[15]_15, 1 for G1, [15] for the determinants expressed in the stigma, _15 for the determinant expressed in the pollen and pollen tubes) 1-[15]_15 × *Dolce Agogia* 2-[23]_2, [44], *Frantoio* 1-45_5 × *Kalamata* 2-[24]_2 [45], *Picholine Marocaine* 2-[24]_2 × *Picholine* 1-[13]_13 [46], and *Arbequina* 1-[13]_13 × *Koroneiki* 2-[26]_6 [47], which were all compatible for DSI and PASI. They did not show selfing in progeny, but they showed contaminants by foreign pollen. Thus, such controlled crosses revealed that selfing is not inherent to the host variety in the presence of double compatible pollen. Selfing appeared when compatible pollen was deficient. Thus, studies in fields do not enable the control of all parameters that may influence selfing. Thus, in production orchards, estimation of the fathers—to conclude on selfing or crossing—would depend on the varieties surrounding each mother, and thus without these records, data should be examined cautiously [43].

### 2.2. DSI First and then PASI Follow One Another

**Figure 3:**
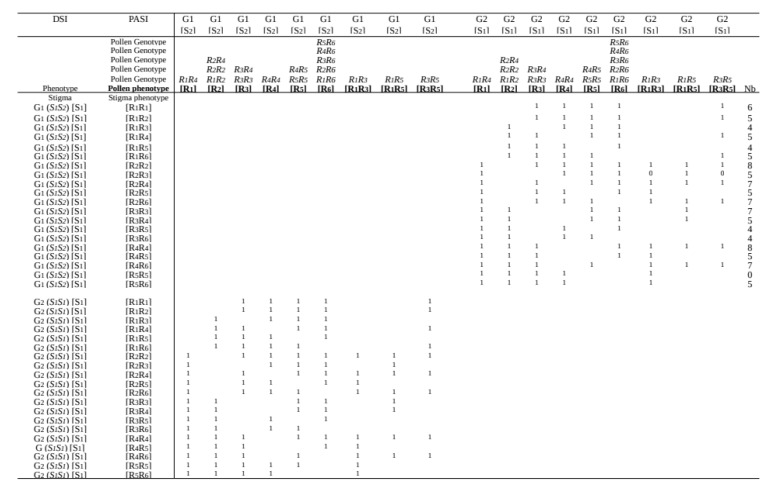
Combination of di-allelic SI (G1 and G2) and poly-allelic SI showing that final fruit success with DSI (1) failed because of PASI (0). Reproduced from Table 1 in Saumitou-Laprade et al. [12] and modified in the frame of DSSM.

To reconcile these disparate results from the literature, we propose that in the olive tree, two different successive control mechanisms of pollen C/I exist, (the dual-successive-screen model, or DSSM). The first one operating at the stigma level is DSI and is classically described in other species of the same Oleaceae family (e.g., Phillyrea) [48]. The second one, PASI, is active at the ovary level, and has not been described yet in Oleaceae. In the olive tree, when a pollen grain germinates on the stigma and the pollen tube grows to the base of the style, this means it did not encounter the incompatibility barrier in these tissues. However, in some cases, because fertilization of one ovule is prevented, this means the incompatibility barrier is triggered somewhere in the transmitting tract tissue between the style and the micropyle [42]. Thus, it would be logical to suppose that the S-genes from PASI, in the female part, would be expressed in the ovary tissue and not in the stigma, as is logical to suppose as the DSI gene does. All observations sustain such a model [1,12,14,16,18]. An interpretation of the outcome of various crosses, according to DSSM, is summarized in Figure 3. The locus D (one of the S_loci) with DSI displays two alleles (*S1S2*) and, thus, each variety belongs either to G1 or G2. The locus P (the second S-locus) with PASI displays six S-alleles.

There are twenty possible female genotypes, but only nine pollen classes, leading to 180 types for stigma phenotype × pollen phenotype (shown in Figure 3) with compatibility (1) or incompatibility (0). In the frame of PASI, so for G1 × G2 and G2 × G1 crosses, considerable data from the literature become logical: asymmetric crosses are explained, such as *Tanche* × *Picholine*, already given, and, as a new example, *Manzanilla* (*R1R2*) × *Arbequina* (*R1R3*) (no fruit because of R1), whereas *Arbequina* × *Manzanilla* (because *R1* is dominated by *R2* in pollen). This occurs in all situations of crosses with *R1R3*, *R1R5*, and *R3R5* crossed with *R1R2*, *R2R3*, and *R2R5*, respectively. Furthermore, it also occurs with *R1R3*, *R1R5*, and *R3R5* crossed with *R1R6*, *R3R6*, and *R5R6*, respectively. However, logically in the frame of DSSM, all crosses should respect DSI. Thus, DSSM is symbolized as 1-1 when pollen germination occurs (DSI) followed by fruit setting (PASI); and as 0-0 when pollen fails to germinate and no fruit setting follows. Consequently, the symbol 1-0 means that pollen has germinated, but fruit set has not occurred. Obviously, the case of 0-1 (fruit set without pollen germination) cannot exist, notwithstanding parthenocarpy, which does not occur in the olive, as male sterile varieties do not produce fruit under bags [49]. Nevertheless, Figure 2 shows that this prediction did not match results from hundreds of crosses examined (see Analyses of Methods and Results from Literature Section 4). Consequently, a mechanism other than SI also operates, allowing self-fertilization.

### 2.3. An Approach with Olive Varieties

**Figure 4:**
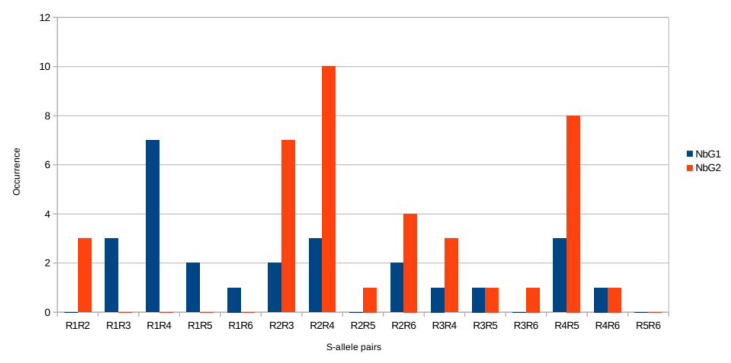
Diagrams showing the frequency of the nine pairs of genotypes in the PASI model and belonging to either a: G1 (S1S2), or b: G2 (S1S1) groups in the frame of the DSI model; raw data are given in Table 3.

Olive growers would probably know whether pollination ensures acceptable production in their orchards. For example, an insufficiency of compatible pollen is suspected when there are seedless shot berries [50], when both normal, as well as underdeveloped, fruits co-occur on a tree. To better understand the situation, it is necessary to calculate the proportion of compatible and incompatible pollen. This is possible when the S-alleles of the varieties are studied [51], allowing relationships to be established between the G groups (G1 or G2) and the S-allele groups attributed to each variety in PASI (Figure 4, Table 4). The six S-alleles are present in G1 and G2 (Figure 3). It is logical to check whether DSI and PASI could work independently. Thus, the data for G1 and G2 are reported, and the PASI pair was added supposing the denominations for varieties corresponded. Table 5 shows that in G1, most varieties carry *R1R3* or *R1R4*, and fewer varieties carry *R2R3*, *R4R5*, or *R2R6*; these pairs are also in G2. Conversely, in G2, most varieties carry *R2R3*, *R2R4*, *R2R5*, or *R2R6*; and, less frequently, some carry *R1R2*, *R3R6*, *R3R4*, or *R3R5*. Consequently, each G1 or G2 group contains pairs with pairwise combinations of the six S-alleles for PASI. Thus, this hypothesis suggests that the D- and P-loci are independent.

### 2.4. Limitation of the Dual-Successive-Screen Model

The case of selfing: The debate on whether or not selfing occurs in olive is now solved, because genotyping the host tree, the father tree, and the embryos determines which one comes from non-self-pollen. Now, the consensus is that SSI works in the olive tree and that selfing occurs in some varieties [43,44,51,52]. Practically, to diagnose SI from cross data, it is necessary to introduce a quantitative threshold and, thus, to accept that the crossing of two incompatible varieties may lead to some fruit. This was perfectly illustrated in the literature in the crosses host *Frantoio* (G1) × *Leccino* (G1) that produced more fruit than in the direction host *Leccino* × *Frantoio* [53]. In the frame of DSSM, the crosses are predicted to fail, but *Frantoio* is more self-fertile than *Leccino* is and, thus, each variety produced fruit by selfing, and the cross is indeed incompatible [11,53]. Pollen tests have never allowed self-compatibility to be revealed, as in *Frantoio* × *Leccino*, because testing was performed the first day after pollen was deposited, although it has been reported that fertilization may occur after 3–5 days (Figure 1) [8,12,13,16,18]. However, it has been shown that pollen tubes make headway from 5 to 10 days [18]. Moreover, erratic fruit set measurements in fruit tests have been commonly documented and widely encountered in the olive tree literature [11], and they were considered to be due to rare events of non-self-pollen entering bags. However, the possibility that selfing may occur should now be considered. In other words, it could be hypothesized that, apparently, the incompatibility barrier is not strict in the stigma and/or ovary tissues.

This weakened incompatibility barrier could have various causes. For example, the S-determinants at the stigma could be short-lived: if the self-pollen can remain alive on the stigma, and thus in the absence of foreign pollen (e.g., under a bag), some pollen tubes could reach the ovaries after 5 days. Indeed, some experimental data sustain that the stigma remains receptive, and that fertilization can occur late, 3–5 days after pollination [8,16,18]. Another possibility is the scarcity of the S-determinants in the ovary. Thus, some pollen tubes may elude recognition and be arrested by the female determinants, and then some ovules could be fertilized by some incompatible pollen. However, the existence of some strictly incompatible crosses is not consistent with this hypothesis. Alternatively, the presence of a modifier loci responsible for self-fertilization has been proposed [54]. Thus, crosses between varieties are predicted to produce at least some fruit resulting from selfing, because modifiers have been shown to be dispersed in the genome and not linked at the S-locus [54].

Finally, it is assumed and retained that degradation of the S-determinant (further DS-D) may occur. Thus, due to DS-D, the SI determinants have become ineffective. Although some pollen tube growth described in field experiments was attributed to “slight differences in the incompatibility responses of the G2 incompatibility group compared to G1”, here, the DS-D hypothesis matches data [29]. Furthermore, the level of self-fertility is S-pair-dependent [42], as the location of the DS-D locus is probably tightly linked to a D or P-locus, in contrast to modifiers that are dispersed in the genome [55]. Interestingly, in most pollen test experiments, the stigmata were fixed about 12–24 h after pollination [12,14], when DS-D is probably not yet operating substantially. Thus, the presence of possible selfing cannot be rejected when only such classical pollen tests are used. Figure 1 shows that, in the olive tree, progression of pollen tubes is slow compared to other species [18]. Moreover, when pollen tubes are allowed to reach the ovary, it has been reported that they accumulate near the ovaries [8,13,16,18]. In production orchards, if such DS-D occurs, and if some flowers are pollinated later, this will not change fruit set. In contrast, under bags, which have isolated inflorescences with only self-pollen, the DS-D could enable some late fruit setting, compatible with observations previously interpreted as bag leakage [12,14,16].

## 3. Recent Findings Argue for Duplication in the Olive Genome

DSSM reconciles pollen and fruit tests. Self-fertilization is made possible because of DS-D. In every cross involving a female carrying *R4R6*, *R4R5*, *R2R4*, or *R2R6*, it must be substantiated that fruit setting is most likely due to pollen of a different variety and not the result of selfing [41]. Moreover, *Rosciola*, *Salonenque* (*R3R5*), *Grossane*, *Leccino* (*R1R5*), *Coucourelle*, *Erbano*, *Aglandau*, *Gordale* (*R2R5*) *Tanche*, *Santa Caterina*, *Bella di Spagna*, and *Konservolia* (*R2R3*) have been reported to be partially self-fertile, a finding that sustains DS-D. In contrast, *R1R3* and *R1R4* genotypes have never been observed to be self-fertile. Thus, the determinants (they are underlined) R2, R4, R5, and R6 should be short-lived in comparison to determinants R1 and R3.

DS-D in PASI is dependent on the S-allele [23]. Two S-loci in olive have been hypothesized [8] and, recently, it was shown that Asterids have undergone paleopolyploidy events [55], and there are molecular hints that S-loci are still duplicated. The seed-specific Fad2 gene is expressed in the embryo (Asterids), which has been found to be duplicated both in olive and sunflower with a normal copy and a copy with the same SiRNA [56,57]. Supposing that the S-locus was duplicated in *Olea*, it is thus likely that the ancestral loci, which functions as DSI in *Phillyrea*, *Fraxinus*, and *Olea*, directs C/I at the stigma/pollen interaction, which was found in all C/I systems [2,8]. The duplicated S-locus has then evolved toward a poly-allelic system at the ovary level. SSI has appeared several times in the plant tree [2] and in Asterids (Asteraceae and Oleaceae), and, thus, in Oleaceae (*Phillyrea*, *Olea*), it is the first time that, in one species, two loci direct C/I via different mechanisms.

Both DSI and PASI in DSSM explain SI, and DS-D explains selfing [14]. Most data are reconciled and there are no more reasons to oppose DSI and PASI. In particular, the same-size G1 and G2 groups are experimented, but because the frequencies of S-pairs are different in G1 and G2 (Table 3), *Tanche* (G2, *R2R3*), for example, has more pollinizers than does *Santa Caterina* (G1, *R2R3*) because pollen 1_R2 is more frequent than pollen 2_R2. Coming back to the example of *Picholine* pollinating *Tanche* because of DSI, there is no fruit, because of PASI. Furthermore, *Cayon* and *Picholine* are inter-incompatible (G1-G1) and *Cayon* is a pollinizer for *Tanche*. However, DS-D explains selfing in mixture with outcrossing, but to explain that selfing may occur, it must be supposed that DS-D could also affect *S1* and *S2* alleles. The flowers remain receptive for 5–6 days, whereas, in hazelnut, C/I is diagnosed after one night, as fertilization has occurred in 12 h [7]. The selfing rate of a variety is due to its PASI pair, but it is mitigated locally on each flower by the composition of the pollen cloud in compatible and incompatible pollen; this explains its variability range observed in all selfing studies reported in the olive tree [14]. In *Arbequina*/*Koroneiki* orchards, selfing occurred due to the lack of coincidence of blossoming [47].

DS-D and paradoxical diagnosis in paternity tests: Paternity tests have been performed as an alternative to the previous poor identification of C/I groups for olive varieties [14]. The pollen donor (father) is identified using molecular markers, thus without any a priori on the pollen donor. However, some embryos could result from late crosses between incompatible pairs due to DS-D. Thus, in this case, paternity tests lead to the identification of a father that is incompatible with the host variety [58]. For example, paternity tests have revealed *Picholine* as the father for fruit harvested on *Lucques*, and *Frantoio* as the father for fruit harvested on *Leccino* and *Grossane* (see Table 6 for additional examples). The explanation is that pairs are compatible through DSI, and incompatible through PASI, but DS-D worked, leading to fecundation by an incompatible pollen grain, leading to a seed. Such an erroneous diagnosis is frequent [58], and thus, it is suggested to use paternity tests to verify whether pollination in orchards is efficient enough to prevent DS-D (Table S3). Then, in an orchard, after sampling only 10 fully formed embryos per tree [51], if some revealed DS-D, this means that there was not enough compatible pollen. DS-D may also function in other Asterids, such as in sunflower and chicory, where self-fertilization occurs at various levels.

**Figure 5:**
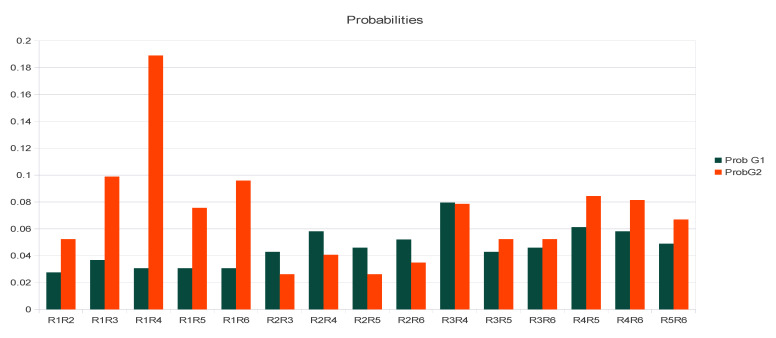
Probability for a variety with a genotype DSI-PASI to match a pollinizer at random in an orchard.

**Figure 6:**
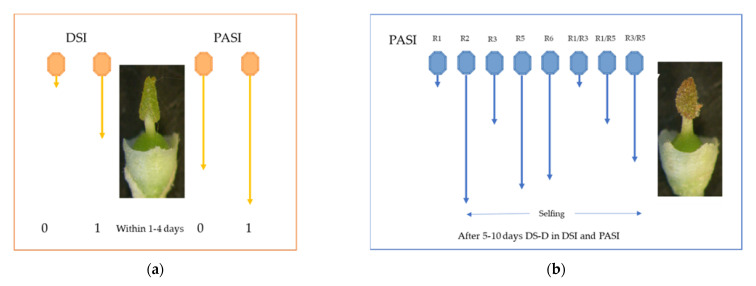
Temporal schemes of the events following pollination (**a**) with compatible pollen within four days, DSI acts, then PASI, leading to 0-0 and 1-0; (**b**) without compatible pollen after 5 to 10 days, DS-D runs for PASI pollen determinants (R1 to R35), leading to pollen tube growth even when incompatible with ovary, but depending upon the S-allele pair in PASI. The length of the blue arrows indicates that the pollen tube growth in selfing is proportional to the risk of DS-D. Pictures are from Adela Olmedilla (Granada, Spain, with permission).

Main features of DSSM: We have shown that two S-alleles in the frame of DSI are insufficient to explain several C/I groups for pairs of olive varieties. Several C/I groups were constructed in the frame of PASI, but pairwise combinations of varieties G1 × G1 and G2 × G2 failed and, thus, PASI alone overestimated mate availability. As, in DSI, G1 × G2 and G2 × G1 crosses are possible only, in PASI, mate availability was overestimated. Data became coherent supposing that two mechanisms for self-incompatibility operate successively in the olive tree, and they match observations of pollen tube germination tests and fruit setting in controlled crosses. The second mechanism, PASI (with at least six alleles detected), appeared probably more recently than DSI, and it functions when pollen tubes have reached the vicinity of the ovary, and thus only when the pollen is compatible through the DSI mechanism. The high rate of self-fertility observed for some individuals (or varieties) is not due to breakage of these mechanisms, but we hypothesize that some S-determinants in the stigma are degraded over time [8,13,16,18], allowing some pollen tubes to take advantage and to reach the ovules. Obviously, details of this phenomenon remain to be studied when the genes for these determinants are identified. Finally, these three mechanisms (DSI, PASI, and DS-D) plus the andromonoecy [60] of the olive tree complexify SI studies, and explain why there have been so many unsettled issues throughout the years of research. The probability for a main variety to match by chance a pollinizer in one orchard is S-pair-dependent (Figure 5) and far from 0.5 [14]. Figure 6 summarizes how DSI first and then PASI operate sequentially and displays the timing to detect the DS-D effect, which infers that compatible pollen is lacking. Selfing is thus incidental due to pollen cloud composition and not inherent to the genotype.

Recently, locus G was mapped in olive linkage group 18 [61], and located on the wild olive genome [56]. Based on the archaeploid model, it can be predicted that PASI will be mapped on the homologous linkage group of LG 18. Functional divergence between sub-genomes following genome duplication is a documented evolutionary mechanism that could explain how two variant systems for SI maintain and function in the olive tree [62].

## 4. Analyses of Methods and Results from Literature

Varieties names provided in Table 1 and Table 2 were extracted from publications dealing with olive cross studies. The database OlSiFaComp was constructed with the variety name of origin in the publication in lines [63]. For each variety, one line corresponds to one bag notations, one open pollination observation, or to one pollen experiment. Thus, for one variety, the repeatability of data in selfing and crosses is immediately available in the table. On 28 May 2021, the table displays 6146 lines. For each line, the original data from the publication are reported in columns. Readers will find in each publication the details of data, the methods used, and the number of fruit or embryos recorded from bags or from paternity tests. Notations were performed based on the original publication and data were completed when feasible by calculations of indices due to andromonoecy and variation in inflorescence structure. The different ways to determine pollen germination and fruit setting are recorded in different columns.

As some international varieties were used in different studies under their local names, although synonyms (*Frantoio*, *Taggiasca*, *Cailletier*, …and others), we kept their original denomination by each author, but in column ‘synonym,’ we can visualize with the button ‘sort’ all the studies performed on one genotype.

For *Manzanilla*, because two different S-allele pairs were deciphered, in all studies with *Manzanilla* as the host, we recorded two lines, one for *R1R2* and one for *R2R4*. As a male, the two *Manzanilla* display the same pollen determinant R2.

This poses the question of the reference of trees in all studies: most authors have their own collections, but some studies were based on international collections, for example, *Grossane* [14] is referred in the collection in Perugia, and the varietal collection is managed by the research unit of Tree Science of the Department of Agricultural, Food and Environmental Sciencesd of the University of Perugia, Italy; thus, it is not the *Grossane* used by Moutier in Montpellier [22]. The comparative study remains to be performed.

In 2012, the PASI alleles were introduced. It appeared that varieties with the same PASI pair behave differently when crossed with one given male. This revealed to us that PASI was insufficient to explain SI, and this justified the fusion of DSI and PASI in the model DSSM.

In 2017, the G group was added and the P alleles were deduced, if possible, and they were deciphered by pollen and bag tests. All synonymies given in publications are reported, but there is no guarantee that the name is sufficient to declare one genotype for varieties sharing one denomination. The database OlSiFaComp displays the lines sorted for varieties identified in G1 or G2 from pollen tests [63].

The coding of varieties in some publication (as Oit, for example) complexified the studies, and it appeared that in different studies, the coding was erroneously pursued as for Koroneiki (Oit55), but also Oit52 in some publications.

In 2020, DS-D was introduced. For all methods used in the studies, we refer readers to original publications because many details in the protocols may vary in pollen tests and seed tests depending on publications. The database contains data on all *Olea europaea* taxa.

## 5. Conclusions

In contradiction of previous assertions, it is suggested that neither DSI nor PASI can predict fruit set alone, because PASI works only when the DSI screen has operated. The two mechanisms could coexist because of paleopolyploidy events that have led to two SI loci (D and P) in the olive genome. Therefore, we propose the dual-successive-screen model (DSSM) supposing two different loci for the expression of the two SI mechanisms. Pollen/stigma is controlled by diallelic SI, or DSI, inferring two G1 and G2 compatibility/incompatibility (C/I) groups for varieties, whereas pollen tubes in ovaries are controlled by poly-allelic SI or PASI with twenty C/I groups.

To explain the selfing of varieties, we suggest that some determinants in the pollen tube and stigma are unstable and degrade (DS-D for degradation of S-determinant) after three to five days, enabling some pollen tubes to avoid being rejected, hence reaching ovules.

Olive growers could have a practical test to reliably identify pollinizers for host varieties. We have shown that paternity tests widely used to diagnose fathers could be used to diagnose DS-D that reveals the lack of compatible pollen (this requires having deciphered varieties both for DSI and PASI). Detection of DS-D means that compatible pollen is insufficient, and thus, planting suitable pollinizers would improve production. The DSSM and DS-D hypotheses are proposed with the hope that they will arouse discussion and motivate further experiments.

## Figures and Tables

**Table 1:** Crosses between host varieties G1 × pollen source G1 reported to have given fruits or embryos.

Host Variety	Pollen Source	References
*Arbequina*	*Cayon*, *Salonenque*, *Giarraffa*, *Nocellara Messinese*	[22,38]
*Ascolana*-*Semi Tenara*	*Picholine*, *Moresca*	[11]
*Ascolana*-*Tenera*	*Picholine*, *Giarraffa*, *Gordal Sevillano*, *Itrana*, *Leccino*, *Moresca*, *Picholine*, *Santa Caterina*	[11]
*Belgentier*	*Cayon*	[22]
*Brun*	*Cayon*	[11]
*Cailletier*	*Selfing*	[11]
*Cayon*	*Cornicabra, Brun*	[11]
*Cornicabra*	*Cornicabra*	[22]
*Frantoio*	*Selfing*	[31,32]
*Frantoio*	*Moraiolo*	[10,25]
*Giarraffa*	*Selfing, Gordales, Nocellara Messina, Picholine, Leccino, Grossa di Spagna, Santa Caterina*	[11]
*Grossane*	*Giarraffa, Grossa di Spagna, Cayanne*	[11]
*LeccinoOit27*	*Moraiolo, Moraesca, Santa Caterina, Giarraffa, Gordales, Frantoio*	[32,39]
*Moresca*	*Selfing, Leccino, Sorani*	[11]
*Picholine*	*Selfing, Sorani, Moresca, GiarraffaOit4, Leccino, SantaCaterina*	[11]
*Santa Caterina*	*Selfing, Grossa di Spagna, Picholine*	[11]
*Taggiasca*	*Selfing, Leccino, Picholine*	[11,31]

**Table 2:** Crosses between host varieties G2 × pollen source G2 reported in the literature to have given fruits or embryos.

Host Variety	Pollen Source	References
*Aglandau*	*VerdaleH*	[22]
*Amellau*	*VerdaleH*	[22]
*Amygdalolia*	*Koroneiki*	[28]
*Bella di Spagna*	*Selfing, Carolea, Kalamon, Manzanilla, Nocellara Etnea, Tanche*	[10]
*Carolea*	*Nostrale Rigali, Maurino, Nocellara Etnea, Bella di Spagna, DolceAgogia, Itrana, Kalamon, Manzanilla*	[11,19,21]
*Cayet roux*	*Bouteillan*	[10]
*Chemlal x*	*Sigoise, Coratina, Blanquette*	[11,18]
*Coratina*	*Nabali Baladi, Carolea*	[24,32]
*DolceAgogia*	*Istrska Belica*	[27]
*DolceAgogia*	*Selfing, Ascolana Tenera*	[11]
*Itrana*	*Ascolana Tenera, Carolea, Itrana, Carolea, Manzanilla-Per ^1^, Bella di Spagna, Carolea*	[11]
*Konservolia* *KoroneikiOit55* *Konservolia x*	*Amygdalolia*	[35]
*Koroneiki*	*Amygdalolia, Kalamata*	[28]
*Koroneiki*	*Aitana, Arbosana, Erbano, Indemoniata, Arbosana, Biancolilla, Indemoniata, Minuta, Nerba, Piricuddara*	[29]
*Koroneiki*	*Bouteillan*	[26]
*Lucques*	*VerdaleH, Bouteillan*	[21]
*Manzanilla-Per*	*Nocellara Etnea, Maurino, Manzanilla, Sevillano, Maurino*	[11]
*Manzanilla Mtp*	*Aglandau*	[22]
*Mastoidis*	*x Amygdalolia, Kalamata, Koroneiki*	[28]
*Nocellara Etnea*	*Bella di Spagna, ManzanillaFarinelli*	[11]
*Nocellara Messina*	*Bella di Spagna, Nocellara Etnea, Bella di Spagna, Bella di Spagna, Manzanilla-Ita*	[11]
*Nostrale Rigali*	*Selfing, Carolea*	[11]
*Olivière*	*Bouteillan, VerdaleH* ^1^ *, ManzanillaMpt* ^1^ *, Amygalolia, Amellau*	[20,22]
*Pendolino*	*Ascolana Tenera, Carolea, Manzanilla* ^1^ *Per* *Selfing*	[11]
*Picual*	*Selfing, Manzanillo, Pendolino, Maurino, Asolana Tenera, Rosciola, PicholineMarocaine, Kalamata*	[11,23,33,36,37]
*VerdaleH*	*Amellau*	[22]

^1^*Verdale de l’Hérault* = *VerdaleH*, *Manzanilla*. Mtp = Montpellier; Per = Perugia.

**Table 3:** Di-allelic self-incompatibility (DSI) and poly-allelic SI (PASI) models functioning at the pollen tube/stigma and pollen tube/ovary levels in the olive tree. DSI from Saumitou-Laprade et al. [12]; PASI from Breton et al. [10].

Type of Inheritance	S-Allele	Dominance Level	Encountered S-Allele Pairs	Expression Level	Expression of Pollen Tube	Remarks
DSI	S1	Recessive versus S2	S1S1 = G2	Stigma	S1	Di-allelic SI
	S2	Dominant versus S1	S1S2 = G1	data	S2	S2S2 cannot exist
		In stigma S2 > S1				
PASI	R6	More dominant	*R1R6*, *R2R6*, *R3R6*, *R4R6*, *R5R6*	Ovary [R1R6], [R2R6], [R3R6], [R4R6], [R5R6]	R6	R6R6 cannot exist unless R7 > R6 exist
	R2		*R1R2*, *R2R3*, *R2R4*, *R2R5*,	Ovary [R1R2], [R2R3], [R2R4], [R2R5]	R2	R2R2
	R1 = R3 = R5		*R1R4*, *R3R4*, *R4R5*, *R1R3*, *R1R5*, *R3R5*	Ovary [R1R3], [R1R5], [R3R5]	R1, R3, R5, R1R3, R1R5, R3R5	R1, R3, R5, R1R3, R1R5, R3R5
	R4	More recessive		Ovary	R 4	R4R4, not encountered yet

**Table 4:** Occurrences of S-alleles in G1 and G2 groups (raw data).

Compatibility Group ^1^	R 1	R 2	R 3	R 4	R 5	R 6
G1 (S1S2)	8	3	6	8	3	2
G2 (S1S1)	1	15	6	10	3	3

^1^ See Figure 4 for the diagram. The list of varieties can be requested from the corresponding author.

**Table 5:** Occurrence of S-allele pairs from PASI in G1 and G2 groups, relative to the number of deciphered varieties from unpublished data.

Genotype	Number of G1 (S1S2)		Number of G2 (S2S2)		Number of Deciphered Varieties
*R* *1R* *2*	0		3	*Manzanilla-Per*	3
*R* *1R* *3*	3	*Picholine, Arbequina*	0		3
*R* *1R* *4*	7	*Cayon*	0		8
*R* *1R* *5*	2	*Grossane, Leccino*	0		2
*R* *1R* *6*	1	*Barnea*	0		2
*R* *2R* *3*	2	*Santa Caterina*	7	*Tanche*	11
*R* *2R* *4*	3		10	*Bouteillan*	22
*R* *2R* *5*	0		1	*Aglandau*	2
*R* *2R* *6*	2		4	*Koroneiki*	6
*R* *3R* *4*	1		3	*Amellau*	8
*R* *3R* *5*	1	*Salonenque*	1	*Rosciola*	3
*R* *3R* *6*	0		1		3
*R* *4R* *5*	3	*Cailletier, Frantoio*	8	*Erbano*	15
*R* *4R* *6*	1	*Moraiolo*	1	*Istrska belica*	4
*R* *5R* *6*	0		0		2
Total	26		39		92

**Table 6:** Paradoxical diagnosis from paternity tests for different pairwise combinations of varieties in the literature, where DSI and PASI are explained by the dual-successive-screen model (DSSM) with or without degradation of S-determinants (DS-D).

Host Variety G2	Father from Host Variety	Father in DSI	Diagnosis ^1^ in Literature [11,12]	Diagnosis ^1^ with DSI [12,59]	Diagnosis ^1^ with PASI [11]	DS-D Functions	Pollinizer after DSSM and DS-D
*Aglandau*	*Frantoio, Petit Ribier*	G1	Compatible	Accept	Reject	Yes	Reject
*Aglandau*	*Cayet Roux*	G2	Compatible	Reject	Reject	No	Reject
*Bouteillan Olivière, VerdaleH* *Verdale de Millas*	*Aglandau, Bouteillan, VerdaleH, Manzanilla*	G2	Compatible	Reject	Reject	No	Reject
*Lucques, Tanche*	*Aglandau, Bouteillan*	G2	Compatible	Reject	Reject	No	Reject
*Lucques, Tanche*	*Picholine, Arbequina*	G1	Compatible	Accept	Reject	Yes	Reject
*Cayet Roux*	*Aglandau*	G2	Compatible	Reject	Accept	Yes	Reject
Host Variety G1	Father from host variety	Father in DSI					
*Grossane*	*Frantoio, Petit Ribier*	G1	Compatible	Accept	Reject	No	Reject
*Cayanne* *Cayon* *Clermontaise*	*Picholine,* *Arbequina*	G1	Compatible	Rejected	Accept	Yes	Reject
*Picholine, Arbequina*	*Amellau, Picual*	G2	Compatible	Accept	Reject	Yes	Reject
*Frantoio, Petit Ribier*	*Cayet Roux*	G2	Compatible	Accept	Reject	Yes	Reject
*Redouneil, Salonenque*	*Amellau, Frantoio, Petit Ribier*	G1	Compatible	Rejected	Reject	No	Reject

^1^ DSI alone accepts fathers erroneously when host and pollen donors are G1 × G2 or G2 × G1. PASI alone accepts fathers erroneously in pairwise combinations for G1 × G1 and G2 × G2.

## Data Availability

All primary cross data are available in Farinelli et al. (2015) [11].

## Supplementary Materials

The following are available online at https://www.mdpi.com/article/10.3390/plants10091938/s1, Table S1: S-allele pairs leading to practical disturbances in orchards through the dual-successive-screen model: DSI and PASI, Table S2: Pairwise combinations of varieties that lead to fruit set in one direction and no fruit in the other direction (asymmetric crosses) because of dominance relationships between the two S-alleles, Table S3: S pairs leading to inconsistencies between the literature, and DSI and PASI after paternity tests, revealing a pollen class that may suffer degradation of S-determinants (DS-D), causing paradoxical diagnosis to identify pollinizers.
