# Supplementary material for: A Dual-Successive-Screen Model at Pollen/Stigma and Pollen Tube/Ovary Explaining Paradoxical Self-Incompatibility Diagnosis in the Olive Tree—An Interpretative Update of the Literature
Source: Plants (Basel). 2021 Sep 17;10(9):1938. doi: 10.3390/plants10091938 (PMC8466169; doi:10.3390/plants10091938)
Supplement: Supplementary file 1 [file plants-10-01938-s001.zip › plants-1341687-supplementary.pdf]

Table S1. S-allele pairs leading to practical disturbances in orchards through the dual-successive-screen model: DSI and PASI.

A<br>Stigma phenotype G1	B<br>Pollen phenotype G2	C<br>Stigma phenotype G2	D<br>Pollen phenotype G1	Fruit due to self-fecundation in the field	Fruit due to self-fecundation in a bag	Fruit due to crosses
[R1R2]	R1	[R1R4]	R2	AB none; CD none	AB none; CD none	none
[R2R4]	R1	[R1R4]	R2	AB yes; CD yes	AB yes; CD none	yes compatible
[R2R6]	R2	[R1R2]	R2	AB yes; CD none	AB yes; CD none	none
[R1R5]	R3R5	[R3R5]	R1R5	AB none; CD none	AB yes; CD yes	none
[R2R3]	R1R3	[R1R3]	R2	AB yes; CD none	AB yes; CD none	yes compatible
[R2R4]	R6	[R2R6]	R2	AB yes; CD none	AB yes; CD yes	none
[R4R5]	R1R5	[R1R5]	R4R5	AB yes; CD yes	AB yes; CD yes	none
[R4R5]	R2R5	[R2R5]	R4R5	AB yes; CD yes	AB yes ; CD yes	none

Table S2. Pairwise combinations of female (Line) x male (Column) leading to compatibility in one direction and incompatibility in the other direction of the cross (asymmetry). All pair shoulb G1xG2 or G2xG1.

Genotype[1]						***R5R6***					
Pollen		*R2R5*				*R4R6*					
Pollen		*R2R4*				*R3R6*					
Pollen		*R2R3*				*R2R6*					
Pollen	*R1R4*	*R1R2*	*R3R4*	***R4R4***	*R4R5*	*R1R6*	*R1R3*	*R1R5*	*R3R5*		
Phenotypes=<br>*determinants*	R1	R2	R3	R4	R5	R6	R1R3	R1R5	R3R5	Number of	combinations
Stigma										symmetric	asymmetric
[R1R2]		*R1R2*				*R2R6*				7	2
[R1R3]		*R1R2,*<br>*R2R3*				*R1R6, R3R6*				7	4
[R1R4]		*R1R2*				*R1R6*				7	2
[R1R5]		*R1R2,*<br>*R2R5*				***R5R6***				7	4
[R1R6]		*R1R2*				*R1R6*				7	2
[R2R2]						*R2R6*				8	1
[R2R3]			*R3R4*			*R3R6*	*R1R3*		*R3R5*	5	4
[R2R4]						*R2R6*				8	1
[R2R5]					*R4R5*	*R1R6, R5R6*		*R1R5*	*R3R5*	5	5
[R2R6]										9	0
[R3R3]		*R2R3*				*R3R6*				7	2
[R3R4]		*R2R3*				*R3R6*				7	2
[R3R5]		*R2R5,R2R3*								8	2
[R3R6]			*R3R4*							8	1
[R4R4]	*R1R4*	*R2R4*	*R3R4*		*R4R5*	*R4R6*				4	5
[R4R5]		*R2R5*				*R5R6*				7	2
[R4R6]										9	0
[R5R5]		*R2R5*				***R5R6***				7	2
[R5R6]					*R4R5*			*R1R5*	*R3R5*	6	3
										140	46

[1] PASI in stigma [R1] = [R2] = [R3] = [R4] = [R5] = [R6] and in pollen *R6 > R2, > R1 = R3 = R5 > R4*

Table S3. This is a table. S -allelepairs leading to inconsistencies between literature, DSI and PASI after paternity tests revealing a pollen class that may suffer degradation of S determinants (DSD) causing paradoxical diagnosis to identify pollinizers

Stigma G1 or G2	Father G2 or G1	Diagnosis in literature	After DSI	After PASI	DSD may	Final Paternity test diagnosis accepted/rejected
R1R2	R1, R1R3,R1R5	Pollinizer	Pollinizer	Rejected	yes	rejected
R1R3	R1, R3, R1R3, R1R4, R1R5, R1R6, R3R4, R3R5, R3R6	Pollinizer	Pollinizer	Reject	yes	rejected
R1R4	R1, R1R3,R1R5	Pollinizer	Pollinizer	Reject	yes	rejected
R1R5	R1, R2, R1R5, R3R5	Pollinizer	Pollinizer	Reject	yes	rejected
R1R6	R1, R1R3, R1R5, R6	Pollinizer	Pollinizer	Reject	yes	rejected
R2R3	R2, R3,R1R3,R3R5	Pollinizer	Pollinizer	Reject	yes	rejected
R2R4	R2	Pollinizer	Pollinizer	Reject	yes	rejected
R2R5	R2,R1R5, R3R5	Pollinizer	Pollinizer	Reject	yes	rejected
R2R6	R2, R6	Pollinizer	Pollinizer	Reject	yes	rejected
R3R4	R3, R1R3,R3R5	Pollinizer	Pollinizer	Reject	yes	rejected
R3R5	R3,R5, R1R3, R3R5, R3 R5	Pollinizer	Pollinizer	Reject	yes	rejected
R3R6	R3, R1R3, R3R5, R6	Pollinizer	Pollinizer	Reject	yes	rejected
R4R5	R5	Pollinizer	Pollinizer	Reject	yes	rejected
R4R6	R6	Pollinizer	Pollinizer	Reject	yes	rejected
R5R6	R5, R1R5, R3R5, R6	Pollinizer	Pollinizer	Reject	yes	rejected
